# Case Report: EGFR-Positive Early-Stage Lung Adenocarcinoma Transforming to Squamous Cell Carcinoma After TKI Treatment

**DOI:** 10.3389/fonc.2021.696881

**Published:** 2021-06-08

**Authors:** Jiatao Liao, Yuan Li, Chang Liu, Qianqian Long, Jialei Wang

**Affiliations:** ^1^ Department of Medical Oncology, Fudan University Shanghai Cancer Center, Shanghai, China; ^2^ Department of Oncology, Shanghai Medical College, Fudan University, Shanghai, China; ^3^ Department of Pathology, Fudan University Shanghai Cancer Center, Shanghai, China

**Keywords:** lung adenocarcinoma, histological transformation, resistance to gefitinib, *EGFR* mutation, *EGFR* amplification, targeted therapy

## Abstract

The histological transformation from epidermal growth factor receptor (EGFR)-mutated adenocarcinoma (ADC) to squamous cell carcinoma (SCC) after tyrosine kinase inhibitor (TKI) treatment is rare. We present a case of a patient who transitioned from early-stage primary lung ADC with partial squamous differentiation, *EGFR* mutation and amplification, to adrenal gland metastasis as SCC with *EGFR* amplification disappearance 115-months after surgery, during which gefitinib and local radiotherapy were utilized for the metastasis in the right femoral head and mediastinal lymph nodes. This case might indicate a possible mechanism of EGFR inhibition resistance with SCC transition and *EGFR* amplification loss from the initially well-responding ADC, especially those with SCC or partial squamous differentiation. The optimal post-progression therapy for ADC-SCC patients is challenging and further studies are needed.

## Introduction

The development of targeted therapy has significantly advanced the treatment of non-small cell lung cancer patients. Epidermal growth factor receptor (EGFR) tyrosine kinase inhibitors (TKIs) have been well supported as beneficial treatments for *EGFR*-mutated lung cancer patients (1, 2).

Despite an initial favorable response to EGFR-TKIs, patients typically experience disease progression within 9 to 12 months (3). The reported drug resistance mechanisms include, but are not limited to acquisition of the *EGFR* T790M mutation in exon 20, *MET* amplification, *PIK3CA* mutation, *HER2* amplification, and small cell histological transformation (4, 5).

The rare phenomenon of transformation from lung adenocarcinoma (ADC) to squamous cell carcinoma (SCC) during EGFR inhibition treatment has also been described as a possible mechanism involving the acquired resistance to EGFR-TKIs (6–8). Herein, we present a new case of *EGFR*-mutant (exon 19 deletion) and *EGFR*-amplified early-stage lung ADC, in which histology revealed partial squamous differentiation, undergoing transformation into metastatic SCC with the disappearance of *EGFR* amplification subsequent to a long-term standard sequential treatment involving surgery, local radiotherapy, and treatment with the first-generation EGFR-TKI, gefitinib.

## Case Description

A 54-year-old man with no smoking history presented with persistent cough in January 2011. Thoracic computed tomography (CT) demonstrated a pulmonary left lower lobe mass. The patient underwent a left lower lobectomy with mediastinal lymph node dissection. The tumor size was 4.0 × 3.5 × 3.5 cm with no lymph node metastasis (pT2aN0M0) and was histologically diagnosed as an adenocarcinoma. No adjuvant chemotherapy or radiotherapy was performed.

**Figure 1:**
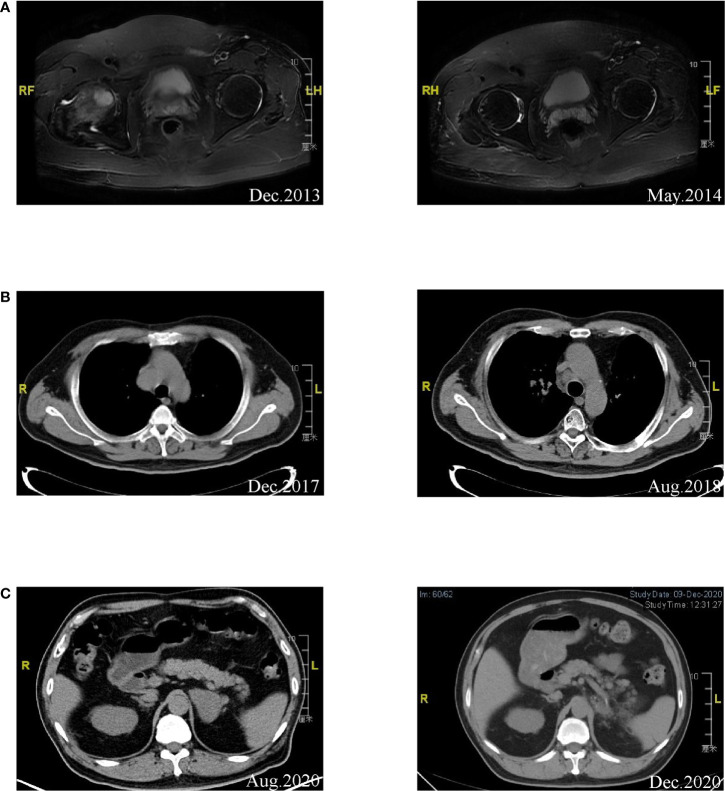
**(A)** Left: MRI of right femoral head indicating metastasis diagnosis. Right: A partial response was achieved after radiotherapy and gefitinib treatment. **(B)** Left: Disease progression was found in mediastinal lymph nodes by CT. Right: A partial response was achieved after continued gefitinib treatment and radiotherapy. **(C)** Left: Metastasis was detected in the left adrenal gland. Right: CT image of the patient after left adrenalectomy was performed.

Two years after the operation, metastasis in the right femoral head was detected by magnetic resonance imaging (MRI) (**Figure 1A**). A deletion at exon 19 of the *EGFR* gene was identified by Sanger sequencing in a surgical specimen of the primary lung carcinoma. The patient started radiotherapy for the right femoral head metastasis followed by gefitinib treatment with 250 mg/day in December 2013 and showed a partial response. Mediastinal lymphadenopathy was detected by CT in December 2017 (**Figure 1B**). There was no disease progression to other sites. The patient refused endobronchial ultrasound-guided needle aspiration (EBUS-TBNA) of the mediastinal masses. Given the absence of *EGFR* T790M mutation detection in the liquid biopsy sample, he continued with gefitinib treatment and received radiotherapy for the mediastinal lymph nodes in January 2018. A partial response was achieved.

**Figure 2:**
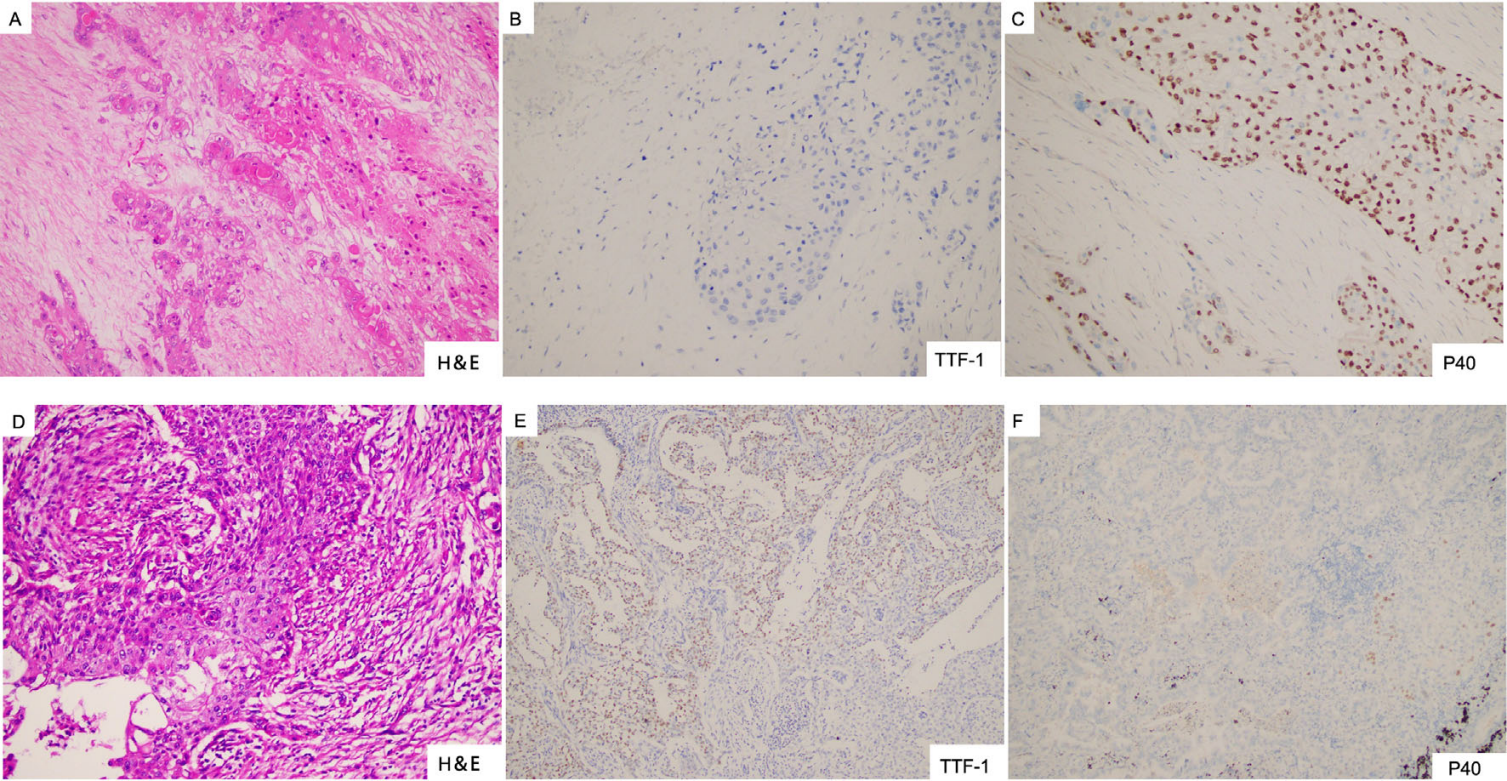
**(A–C)** Left adrenal gland specimen showing squamous cell carcinoma with negative staining for TTF-1 and partially positive staining for P40. **(D–F)** Resected lung sample showing adenocarcinoma with partial squamous differentiation, expressing focally positive TTF-1 and P40.

Disease progression was revealed by CT showing a metastasis in the left adrenal gland in August 2020 (**Figure 1C**). The patient then received treatment with the third-generation EGFR-TKI almonertinib at 110 mg/day in August 2020 and showed stable disease. Left adrenalectomy was performed in October 2020. Squamous cell carcinoma histology was identified from the left adrenal gland specimen by immunohistochemistry, which was TTF-1 and NapsinA negative, P40 partially positive, and CK7 positive (**Figures 2A–C**). The sample was subjected to a sequencing study using FoundationOne CDx (an FDA-approved 324-gene panel assay), and the results showed an *EGFR* exon 19 deletion (delE746_A750, allele frequency [AF]: 26.68%). Histopathological re-examination of the surgical sample from the resected primary lung carcinoma revealed adenocarcinoma with partial squamous differentiation (less than 5%), which was partially positive for TTF-1, NapsinA, and CK5/6, focally positive for P40, and CK7 positive (**Figures 2D–F**). Sequencing results of the initial lung specimen demonstrated the same *EGFR* 19del (delE746_A750, AF: 80.37%) and *EGFR* amplification (copy number [CN]: 30).

**Figure 3:**
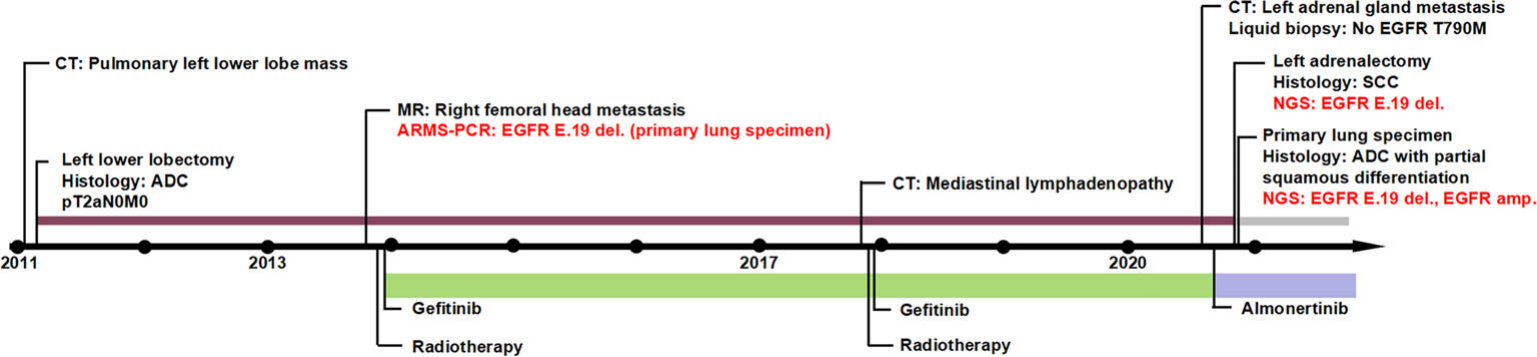
Timeline of the clinical course.

The timeline illustrating this patient’s medical history and treatment is presented in **Figure 3**. The patient is currently continuing almonertinib monotherapy and the disease is stable.

## Discussion

The mechanisms underlying of ADC-SCC transformation during treatment with EGFR inhibitors remain unclear. A possible explanation is that ADC and SCC co-existed in the original tumor and only the SCC component progressed following after EGFR-TKI treatment (9). In this case, pathological and immunohistochemical results revealed the transformation from lung adenocarcinoma with partial squamous differentiation to metastatic SCC, which may support this assumption. There is also a possibility that pluripotent tumor stem cells acquire a divergent phenotype under the pressure of TKI (7). We considered a second primary tumor unlikely because the original *EGFR* deletion in exon 19 was maintained following the SCC transition in this patient.

Previous studies show that, *live kinase B1 (LKB1)* inactivation can promote gradual transition from lung ADC to SCC in mouse model (10), leading to drug resistance through metabolic alteration (11). Transition of ADC-SCC with disease progression was also observed in lung cancer patients treated with chemotherapy and immunotherapy (12, 13), indicating that ADC-SCC transition might be a common drug-resistance mechanism. To better understand the link between ADC-SCC transition and EGFR inhibition resistance, further experimental validation is required.

The simultaneous occurrence of ADC-SCC transformation and EGFR inhibition resistance is rare. To date, there have been only 22 reported cases including our patient, and their characteristics are summarized in **Table 1**. Based on the available information, most patients described were female (72.7%) and the median age was 62 years (range, 40 to 79 years). In most cases, the histological transition was found at the lung recurrence site. This case is unique to present the ADC-SCC transition in the adrenal gland metastasis, which has never been reported before.

**Table 1:** Characteristics of patients with transition to SCC from primary lung ADC after EGFR-TKIs treatment.

Ref	Sex/Age	Stage	Original site		PFS	After transition to SCC				OS	Alive	
			ADC genomic findings	Initial treatment		Relapsed/metastatic site		SCC genomic findings	Subsequent therapy			
1 (6)	F/58	III	EGFR E.19 del.	Erlotinib, surgery, cisplatin/pemetrexed, radiotherapy	11	Lung		EGFR E.19 del., E.20 T790M	NA	20	Yes	
2 (14)	F/63	IV	WT	Carboplatin/pemetrexed, bevacizumab	12	Lung		EGFR E.21 L858R, E.20 T790M	NA	43	NA	
3 (15)	F/51	IV	EGFR E.19 del.	Gefitinib, surgery	10	Lung		EGFR E.19 del.	Gemcitabine/cisplatin	10	Yes	
4 (15)	F/61	IV	EGFR E.21 L858R	Gefitinib	12	Pleura		EGFR E.21 L858R	Erlotinib	24	No	
5 (16)	F/63	IV	EGFR E.21 L858R	Erlotinib	5	Lung		EGFR E.21 L858R, PIK3CA mut.	Gefitinib, carboplatin/gemcitabine	14	No	
6 (7)	F/66	IV	EGFR E.19 del.	Carboplatin/pemetrexed	4	Lung		EGFR E.19 del.	NA	12	No	
7 (17)	F/48	III	EGFR E.19 del.	Adjuvant chemotherapy	NA	Lung		EGFR E.19 del.	NA	30	No	
8 (17)	F/64	IV	EGFR E.21 L858R, E.20 T790M	Gefitinib	NA	Lung		EGFR E.21 L858R, E.20 T790M	Rociletinib	NA	Yes	
9 (18)	F/74	IV	EGFR E.21 L858R	Gefitinib	9	Lung		EGFR E.21 L858R, E.20 T790M	Carboplatin/ vinorelbine	21	Yes	
10 (18)	F/79	IV	EGFR E.19 del.	Gefitinib	17	Lung		EGFR E.19 del., E.20 T790M	Gefitinib	26	Yes	
11 (19)	F/44	IV	EGFR E.19 del.	Afatinib, radiotherapy, denosumab	18	Lung		EGFR E.19 del., E.20 T790M	Osimertinib, radiotherapy	21	Yes	
12 (20)	F/67	IV	EGFR E.19 del.	Afatinib	6	Cervical lymph node		EGFR E.19 del., PIK3CA mut.	Afatinib, platinum/vinorelbine	10	Yes	
13 (21)	F/43	IV	EGFR E.21 L858R	Gefitinib, radiotherapy	8	Lung		EGFR E.21 L858R, E.20 S768I	Gefitinib	11	No	
14 (22)	M/40	I	EGFR E.19 del.	Surgery, vinorelbine/cisplatin	48	scalp		EGFR E.19 del., E.20 T790M	NA	72	NA	
15 (23)	M/68	I	EGFR E.21 L858R	Surgery, tegafur/uracil	48	liver		EGFR E.21 L858R, E.20 T790M	Osimertinib	77	No	
16 (24)	F/52	IV	EGFR E.19 del.	Erlotinib, bevacizumab	12	muscle		EGFR E.19 del.	Docetaxel, radiotherapy, afatinib	29	NA	
17 (25)	M/62	I	EGFR E.21 L858R	Surgery, tegafur/uracil	6	Pleura		EGFR E.21 L858R	Cisplatin/pemetrexed	16	No	
18 (26)	F/67	IV	EGFR E.21 L858R, E.20 T790M	Gefitinib	58	Lung		EGFR E.21 L858R	Carboplatin/gemcitabine	70	No	
19 (27)	F/56	III	EGFR E.19 del.	Surgery, platinum doublet, gefitinib	72	Lung		EGFR E.19 del., E.20 T790M	Osimertinib, surgery	114	No	
20 (28)	M/61	IV	EGFR E.19 ins.	Erlotinib	28	Lung		EGFR E.20 T790M, PTEN mut.	Osimertinib, pembrolizumab, S-1	58	No	
21 (28)	M/72	IV	EGFR E.21 L858R	Erlotinib	9	Lung		EGFR E.21 L858R, E.20 T790M, PTEN mut. TP53 mut.	Osimertinib, pembrolizumab	31	No	
22	M/54	I	EGFR E.19 del., EGFR amp.	Surgery	24	Adrenal gland		EGFR E.19 del.	Almonertinib, surgery	123	Yes	

ADC, adenocarcinoma; Alive, alive at the last follow-up; EGFR-TKI, epidermal growth factor receptor-tyrosine kinase inhibitor; NA, not available; OS, overall survival from ADC diagnosis; PFS, progression-free survival from initial treatment; SCC, squamous cell carcinoma.

According to **Table 1**, approximately half of the patients developed *EGFR* T790M as an acquired resistance mechanism to EGFR-TKI therapy. Other genomic alterations included the acquired mutations in *EGFR* S768I, *PIK3CA*, *PTEN*, *TP53*. We describe the first reported histologic evolution of ADC to SCC combined with the disappearance of *EGFR* amplification.

In this case, sequencing analysis of the primary lung carcinoma demonstrated *EGFR* deletion in exon 19 (AF: 80.37%) and *EGFR* amplification (CN: 30). There is evidence indicating that lung ADC patients with higher *EGFR* mutation abundance benefit more from EGFR-TKIs (29). Further, high *EGFR* copy number has been associated with better clinical outcomes in *EGFR*-mutant patients treated with EGFR-TKI (30, 31). Studies have shown that *EGFR* amplifications usually impact mutated but not wild-type alleles (31), which is likely to increase *EGFR* mutation abundance and render cancer cells more sensitive to EGFR inhibition. These may explain why the effectiveness of gefitinib and local radiotherapy was sustained for up to 48 months for the first metastasis in the right femoral head, and up to 32 months for the second metastasis in the mediastinal lymph nodes.

A limitation of this report was that no biopsy of the lesions was performed for the first and second metastases. Therefore, we cannot exclude the assumption that ADC-SCC transition had already occurred before the detection of the adrenal gland metastasis.

*EGFR*-mutated patients resistant to TKIs with a changed phenotype to SCC show poor prognosis with a median overall survival of only 3.5 months (26). The management of transformed SCC after TKI resistance is controversial. According to **Table 1**, selected treatment strategies include combining chemotherapy or radiotherapy, surgery, third-generation TKI, and immunotherapy. To date, this patient has been treated with almonertinib for 8 months with stable disease. Further studies in *EGFR*-mutant patients with TKI resistance and ADC-SCC transformation are needed to specify the underlying mechanisms and to optimize the individualized post-progression therapy.

## Data Availability Statement

The original contributions presented in the study are included in the article/supplementary material. Further inquiries can be directed to the corresponding author.

## Ethics Statement

Ethical review and approval were not required for the study on human participants in accordance with the local legislation and institutional requirements. The patients/participants provided their written informed consent to participate in this study.

## Author Contributions

JW and JL designed the study drafted the manuscript. YL and CL collected and analyzed the patient data. QL and JL contributed to the literature research. JW reviewed and edited the manuscript. All authors contributed to the article and approved the submitted version.

## Funding

This study was sponsored by the Natural Science Foundation of Shanghai (grant number: 19ZR1410400).

## Conflict of Interest

The authors declare that the research was conducted in the absence of any commercial or financial relationships that could be construed as a potential conflict of interest.

## Abbreviations

AF, allele frequency; ADC, adenocarcinoma; CT, computed tomography; CN, copy number; EGFR, epidermal growth factor receptor; MRI, magnetic resonance imaging; SCC, squamous cell carcinoma; TKI, tyrosine kinase inhibitor.
